# Comparative Toxicity of Nanoparticulate CuO and ZnO to Soil Bacterial Communities

**DOI:** 10.1371/journal.pone.0034197

**Published:** 2012-03-29

**Authors:** Johannes Rousk, Kathrin Ackermann, Simon F. Curling, Davey L. Jones

**Affiliations:** 1 Environment Centre Wales, Bangor University, Gwynedd, United Kingdom; 2 Section of Microbial Ecology, Department of Biology, Lund University, Lund, Sweden; 3 BioComposites Centre, Bangor University, Gwynedd, United Kingdom; Missouri University of Science and Technology, United States of America

## Abstract

The increasing industrial application of metal oxide Engineered Nano-Particles (ENPs) is likely to increase their environmental release to soils. While the potential of metal oxide ENPs as environmental toxicants has been shown, lack of suitable control treatments have compromised the power of many previous assessments. We evaluated the ecotoxicity of ENP (nano) forms of Zn and Cu oxides in two different soils by measuring their ability to inhibit bacterial growth. We could show a direct acute toxicity of nano-CuO acting on soil bacteria while the macroparticulate (bulk) form of CuO was not toxic. In comparison, CuSO_4_ was more toxic than either oxide form. Unlike Cu, all forms of Zn were toxic to soil bacteria, and the bulk-ZnO was more toxic than the nano-ZnO. The ZnSO_4_ addition was not consistently more toxic than the oxide forms. Consistently, we found a tight link between the dissolved concentration of metal in solution and the inhibition of bacterial growth. The inconsistent toxicological response between soils could be explained by different resulting concentrations of metals in soil solution. Our findings suggested that the principal mechanism of toxicity was dissolution of metal oxides and sulphates into a metal ion form known to be highly toxic to bacteria, and not a direct effect of nano-sized particles acting on bacteria. We propose that integrated efforts toward directly assessing bioavailable metal concentrations are more valuable than spending resources to reassess ecotoxicology of ENPs separately from general metal toxicity.

## Introduction

Manufactured particles with at least one dimension between 1 and 100 nm [1] have been termed Engineered nanoparticles (ENPs). Metal oxide ENPs are receiving increasing attention in material science and nano-technology based industries for a large variety of applications, including catalysts, sensors and for their incorporation into commercial products [2]. For instance, ENP CuO is used in semiconductors, catalysts and in photovoltaic cells [3], while ENP ZnO is used in personal care products as well as coatings and paints [4] due to its UV absorption efficiency and transparency to visible light that increases with smaller particle size. The increasing industrial application of ENPs is likely to increase their environmental release, especially exposing soils and freshwaters [4], [5], prompting a careful evaluation of their ecotoxity in this environment. The small size of the ENP, and the greater mobility and potentially increased risk of uptake by organisms that this confers, have been proposed to increase the toxic potential of ENP substances generally, as demonstrated *in vitro* [6]. Several studies have demonstrated the potential of metal oxide ENPs as environmental toxicants [7], [8]. However, lack of suitable control treatments have compromised the power of early assessments to determine and quantify the potential environmental impact in a useful context, i.e. whether the ENP property of the substance made it more toxic than it would have been in a generic (non-ENP) form. To enable progress in our understanding of the ecotoxicology of ENPs, it is important to assess (i) whether the nano-form size that characterizes ENP *per se* increases the toxicity beyond other forms of the substance, and (ii) how the observed toxicity of the ENP compares to the well-known toxicity of ionic forms of heavy metals. That is, while many studies have been conducted to determine if heavy metal ENPs can be toxic, there is a scarcity of studies that have investigated if the ENP form of heavy metal toxicants are more toxic than non-ENP forms of the same substance.

Here, we evaluated the ecotoxicity of ENP forms of Zn and Cu oxides in two different soils. In addition to the ENP forms of the metal oxides, we also included two reference toxicant forms, (i) bulk oxide of non-nanoparticulate form, and (ii) highly soluble sulfate forms of the metals. The bulk form of the metal oxide is designed as a control to show if any observed toxicity was due to its nano-particulate form, while the soluble sulfate form acts as a control to elucidate the extent to which observed toxicity is due to metal ion solubilization in soil solution. By measuring the resulting metal concentrations in soil solution, we strengthen the connection between metals and toxicity. To provide a sensitive measure of ecotoxicity we measured the effect of the substance additions on bacterial growth using the leucine incorporation method [9], [10], previously successfully used to accurately determine toxicity of environmental toxicants including metals [11], [12], antibiotics [13]–[15], phenols [16] and salt [17].

## Materials and Methods

### Soils and chemicals

We used two different soils for the experiment, one mineral pasture soil (Typic Dystrochrept, organic-C = 40 mg g^−1^, total-N = 3.3 mg g^−1^, pH(H_2_O) = 5.0; henceforth “mineral soil”) and one organic pasture soil (Typic Fragiochrept, organic-C = 154 mg g^−1^, total-N = 9.3 mg g^−1^, pH(H_2_O) = 6.6; henceforth “organic soil”). These soils are described in detail elsewhere [18]. Soils were fresh sieved (<2 mm), and then adjusted to a moisture content of 40% of water holding capacity (WHC) for the mineral soil, and 60% WHC for the organic soil, both deemed to be optimal for microbial activity based on previous work. After these preparations the soils were incubated at 20°C for one week before experimentation commenced. The CuO (particle size 40–80 nm; henceforth “nano-CuO”) and ZnO (particle size 20 nm; henceforth “nano-ZnO”) ENPs used in the experiment were supplied and guaranteed by IO-LI-TEC nanomaterials (Heilbronn, Germany) and had CAS reference numbers 1317-38-0 and 1314-13-2, respectively. The bulk forms of the metal oxides and sulfates of the metals were standard laboratory grade chemicals (ZnO CAS: 1314-13-2, henceforth “bulk-ZnO”; ZnSO_4_·7H_2_O CAS: 7446-20-0; CuO CAS: 1317-38-0, henceforth “bulk-CuO”; CuSO_4_·5H_2_O CAS: 7758-99-8, all supplied by Sigma Aldrich, St Louis, USA).

### Experimental design

Subsamples of soil (2.0 g dry weight equivalents) were weighed into 50 ml centrifugation tubes, to which immediately were added 200 mg laboratory grade acid washed sand (40–100 µm mesh) carrying the different toxicants. Nano-ZnO, bulk-ZnO, ZnSO_4_, nano-CuO, bulk-CuO and CuSO_4_ were added at 8 concentrations at logarithmic intervals from 0 to 200 mmol metal g^−1^ soil. The sand-toxicant mixtures were mixed into the soil samples through vigorous shaking and stirring with a clean spatula to ensure homogenous application, and all treatments were run in independent duplicates. The samples were incubated for a period of 5–7 h to allow sufficient mixing and equilibration of the sample, yet sufficiently brief to ensure that the innate soil bacterial tolerance to the toxicant additions, rather than the induced tolerance following the selective growth of a tolerant community [12]–[14], [16], were assessed. After this incubation, all samples were analyzed for bacterial growth using the leucine incorporation method [19] adapted for soil [9], [20], importantly using short incubation periods (2 h). To estimate this, 20 ml water were added to the 2 g soil sample, followed by a homogenization/centrifugation step [9] to extract a bacterial suspension, which subsequently was used to estimate bacterial growth. Subsets of the same suspension were also analyzed for concentrations of Zn and Cu and for pH. After a filtration step (0.45 µm), soil solutions were analysed for metal concentrations using an inductively coupled plasma optical emission spectrophotometer (ICP-OES; Varian 700 – ES, Varian Inc. Scientific Instruments, Palo Alto, USA). Initially a semi-quantitative analysis was performed to determine major constituents using internal calibration to screen for any interference. The target metal ion concentrations were then determined quantitatively by calibration against a series of standard solutions derived from a commercial multi-element standard (Sigma-Aldrich, St Louis, USA) with preparation blanks used to determine background concentrations.

### Particle size characterization

To characterize the particle size of the bulk form metal oxides, 10 g subsamples were added to stainless steel sieves (53 µm grid; a size fraction routinely used to differentiate between particulate and soluble organic matter [21]), that were subjected to shaking on a rotary shaker (200 rpm) overnight (16 h), with filter paper collectors placed below. The fraction of the subsample falling through the sieve was collected and weighed to characterize the proportion of fine particles in the bulk materials.

### Statistical analysis and calculations

Tolerance values were expressed as the logarithm of the concentration of toxicant resulting in 50% inhibition (concentration at 50% effect, EC_50_) of the short term assay for bacterial growth. A more potent toxicant inhibits the bacterial growth at a lower concentration, and thus a lower log(EC_50_) indicates higher toxicity. The log(EC_50_) values were determined by fitting a sigmoidal curve to model the concentration-response relationship, i.e. bacterial growth along the range of added metal concentrations, *y = c/(1+e^b(x−a)^)*, where *y* is the relative bacterial growth, *x* is the logarithm of the added concentration of metal, *c* is the bacterial growth in the no-addition control (at 0 mmol metal g^−1^ soil) *a* is the value of log(EC_50_) and *b* is the parameter indicating the slope of the inhibition curve. The bacterial growth data was normalized to unity to present data as relative bacterial growth. KaleidaGraph 4.0 for Mac (Synergy software) was used to fit the model to the experimental data. To provide a more accurate estimate of bacterial growth in the unamended control (i.e. at 0 mmol g^−1^ added toxicant), the average of all the 0 mmol g^−1^ metal additions (i.e. nano, bulk and sulfate forms of both metals, all being the same treatment) were combined for each soil. The concentration dependence of metal concentrations in soil solution was tested using regression analysis (JMP 9.0 for Mac, SAS Inst., USA).

## Results

### Cu-toxicity for soil bacteria

**Figure 1:**
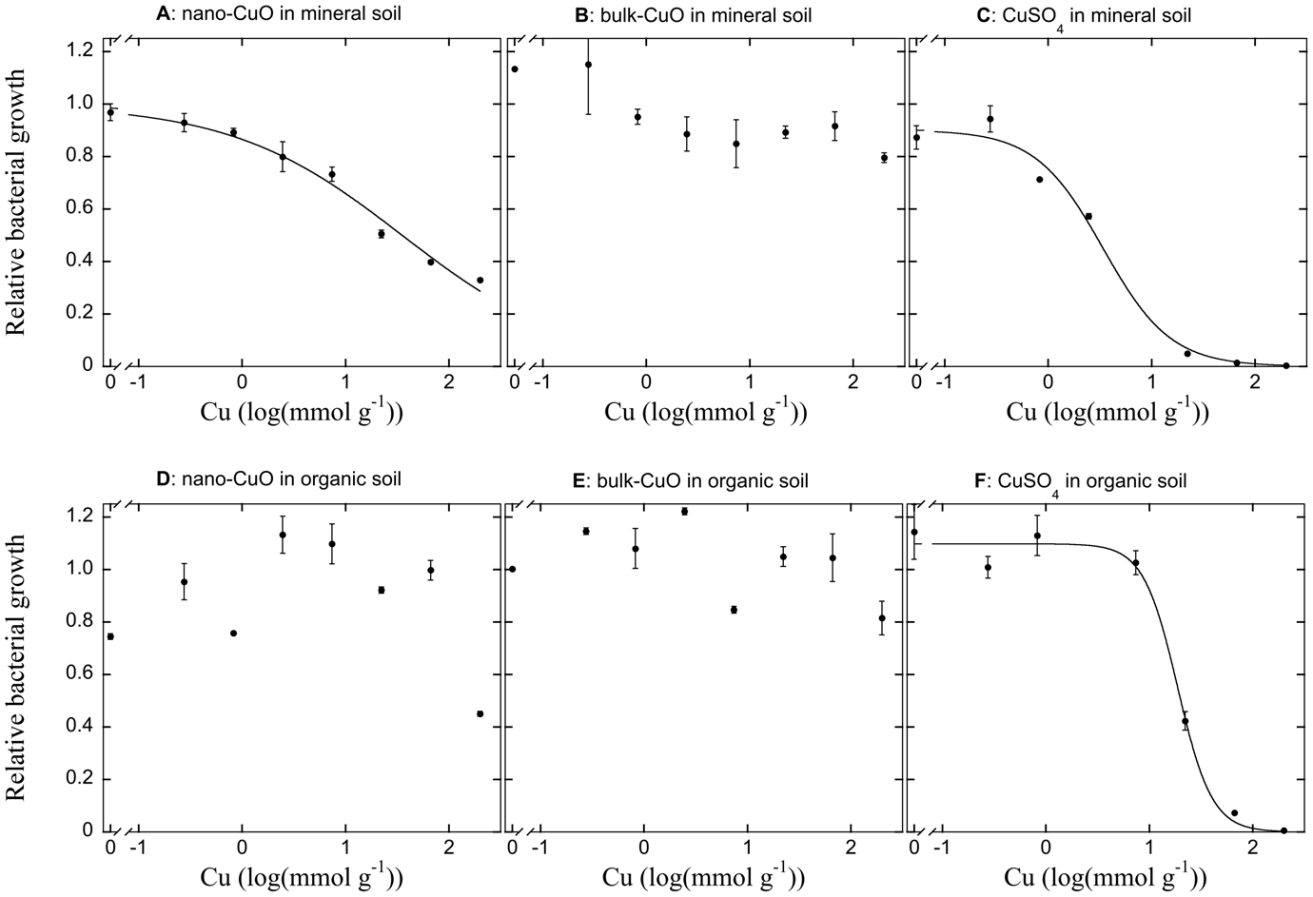
Cu toxicity to bacterial communities in mineral (panels A, B, C) and organic (panels D, E, F) soils. The effects of nano-sized (i.e. ENPs; panels A, D) and macroparticulate ‘bulk-sized’ (i.e. non-ENP) oxide (panels B, E) and as well as sulfate forms of Cu (panels C, F) on soil bacterial community growth rate are contrasted. The relationship between the relative bacterial growth (normalized relative to the bacterial growth rate in unamended soils) and rate of Cu application are described with a sigmoidal curve to establish the concentration response relationship. Only statistically significant relationships are presented as lines. Datapoints represent the mean of two independent replicates ±1 SE.

The toxic effect of CuSO_4_ on bacterial growth was clear in both soil types (Fig. 1C, F), with increasing concentrations of added toxicant effectively reducing bacterial growth to virtually zero, resulting in a tight fit of the sigmoidal curve equation used to establish inhibition curves (R^2^>0.99 for both soils). Using the log(EC_50_) as an index for toxicity of the metals, the bacterial growth was more susceptible to CuSO_4_ in the mineral soil (log(EC_50_) = 0.52±0.09; estimate ±1 SE) than in the organic soils (log(EC_50_) = 1.28±0.04). This suggested that 50% of the bacterial growth was inhibited by about 3.4 mmol CuSO_4_ g^−1^ soil in the mineral soils, and that about 19 mmol CuSO_4_ g^−1^ resulted in a similar inhibition of bacterial growth in the organic soil. Bulk-CuO appeared inert by comparison, and did not appear to affect the bacterial growth in either soil in the studied interval (Fig. 1B, E). In contrast, the ENP form of CuO produced a pronounced inhibition of bacterial growth in the mineral soil (Fig. 1A), resulting in a clear concentration response curve that could be well-described by a sigmoidal model (R^2^ = 0.98), and thus proved toxic to the bacterial community (log(EC_50_) = 1.55±0.10). The toxic effect of the nano-CuO was much less pronounced in the organic soil (Fig. 1D), and we could not see clear evidence for a concentration-response relationship. However, we note that the highest concentration of nano-CuO (200 mmol g^−1^) did appear to suppress bacterial growth somewhat in the organic soil.

### Zn-toxicity for soil bacteria

**Figure 2:**
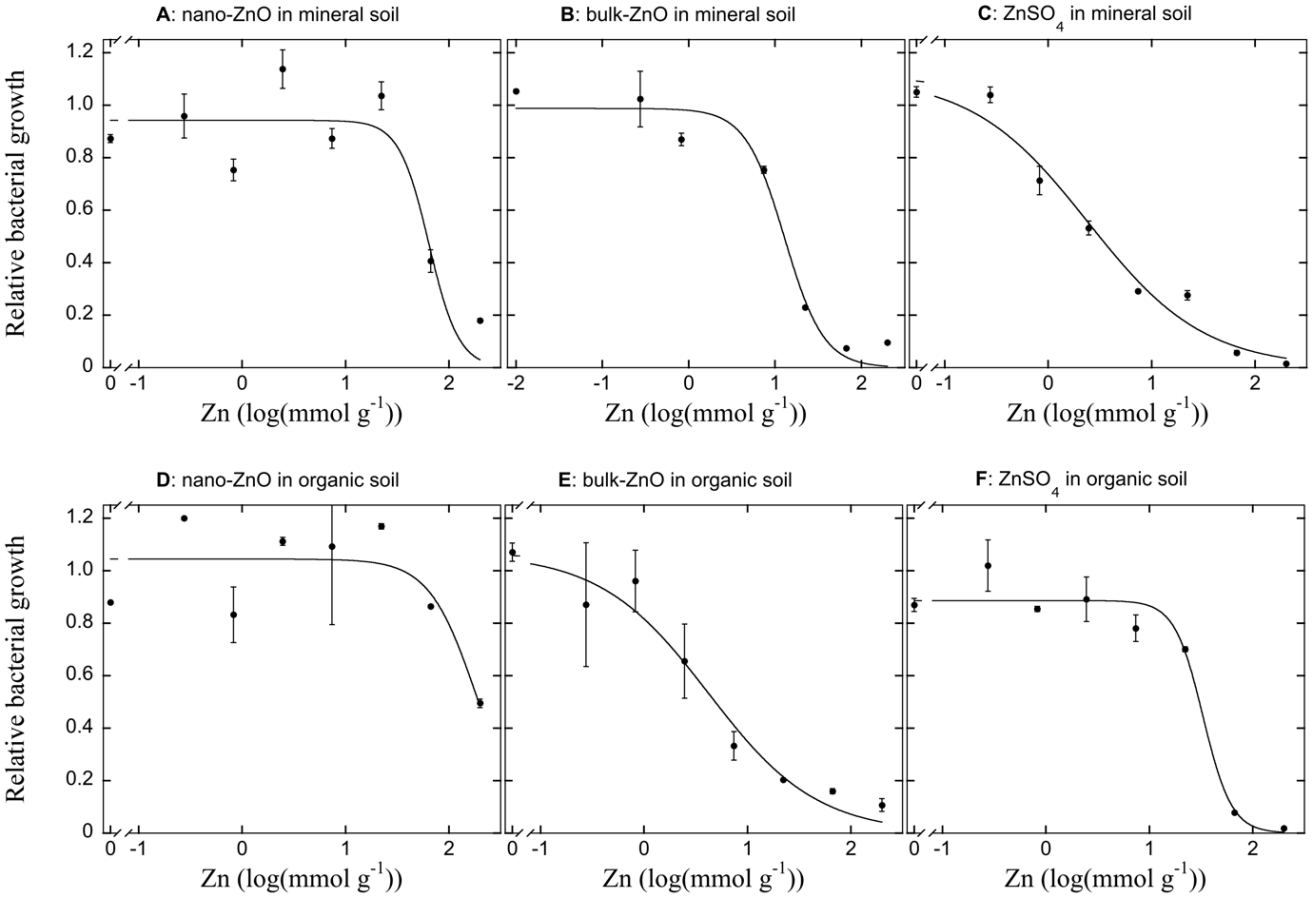
Zn toxicity to bacterial communities in mineral (panels A, B, C) and organic (panels D, E, F) soils. The effects of nano-sized (i.e. ENPs; panels A, D) and macroparticulate ‘bulk-sized’ (i.e. non-ENP) oxide (panels B, E) and as well as sulfate forms of Zn (panels C, F) on soil bacterial community growth rate are contrasted. The relationship between the relative bacterial growth (normalized relative to the bacterial growth rate in unamended soils) and rate of Zn application are described with a sigmoidal curve to establish the concentration response relationship (presented as lines). Datapoints represent the mean of two independent replicates ±1 SE. Sometimes error bars are hidden by symbols.

The toxic effect of ZnSO_4_ on bacterial growth was clear in both soil types (Fig. 2C, F), and increasing concentrations of added toxicant effectively suppressed bacterial growth to virtually zero, resulting in a good fit of the sigmoidal curve used to model the concentration-response relationships (R^2^>0.97 for in both soils). Using the log(EC_50_) as an index for toxicity of Zn, the bacterial growth was more susceptible to ZnSO_4_ in the mineral soil (log(EC_50_) = 0.38±0.15) than in the organic soil (log(EC_50_) = 1.52±0.07). This suggested that 50% of the bacterial growth was inhibited by about 2.4 mmol ZnSO_4_ g^−1^ soil in the mineral soil, and that about 33 mmol ZnSO_4_ g^−1^ resulted in a similar inhibition in the organic soil. Bulk-ZnO also effectively inhibited the bacterial growth in both soils in the studied interval (Fig. 2B, E), and clear concentration-response relationships could be estimated (both R^2^>0.97; Fig. 2B, E). Using the log(EC_50_) as an index for potency of the substances in the two soils, it was evident that bulk-ZnO was more toxic to bacteria in the organic soil (log(EC_50_) = 0.62±0.15) than in the mineral soil (log(EC_50_) = 1.11±0.08). This suggested that 50% of the bacterial growth was inhibited already by 4.2 mmol bulk-ZnO g^−1^ soil in the organic soils, and that the same inhibition of bacteria only occurred by 13 mmol bulk-ZnO g^−1^ in the mineral soil. The ENP forms of ZnO also effectively reduced bacterial growth in both soils, and thus we could establish significant concentration-response relationships in both mineral (R^2^ = 0.83; Fig. 2A), and in the organic soils (R^2^ = 0.68; Fig. 2D). The nano-ZnO appeared to inhibit the bacterial communities more effectively in the mineral soil (log(EC_50_) = 1.81±0.10) than in the organic soil (log(EC_50_) = 2.27±0.14). This suggested that 50% of the bacterial growth was inhibited by about 64 mmol nano-ZnO g^−1^ soil in the mineral soil, whilst the same inhibition was only reached at 185 mmol Zn g^−1^ soil in the organic soil.

### Cu in soil solution

**Figure 3:**
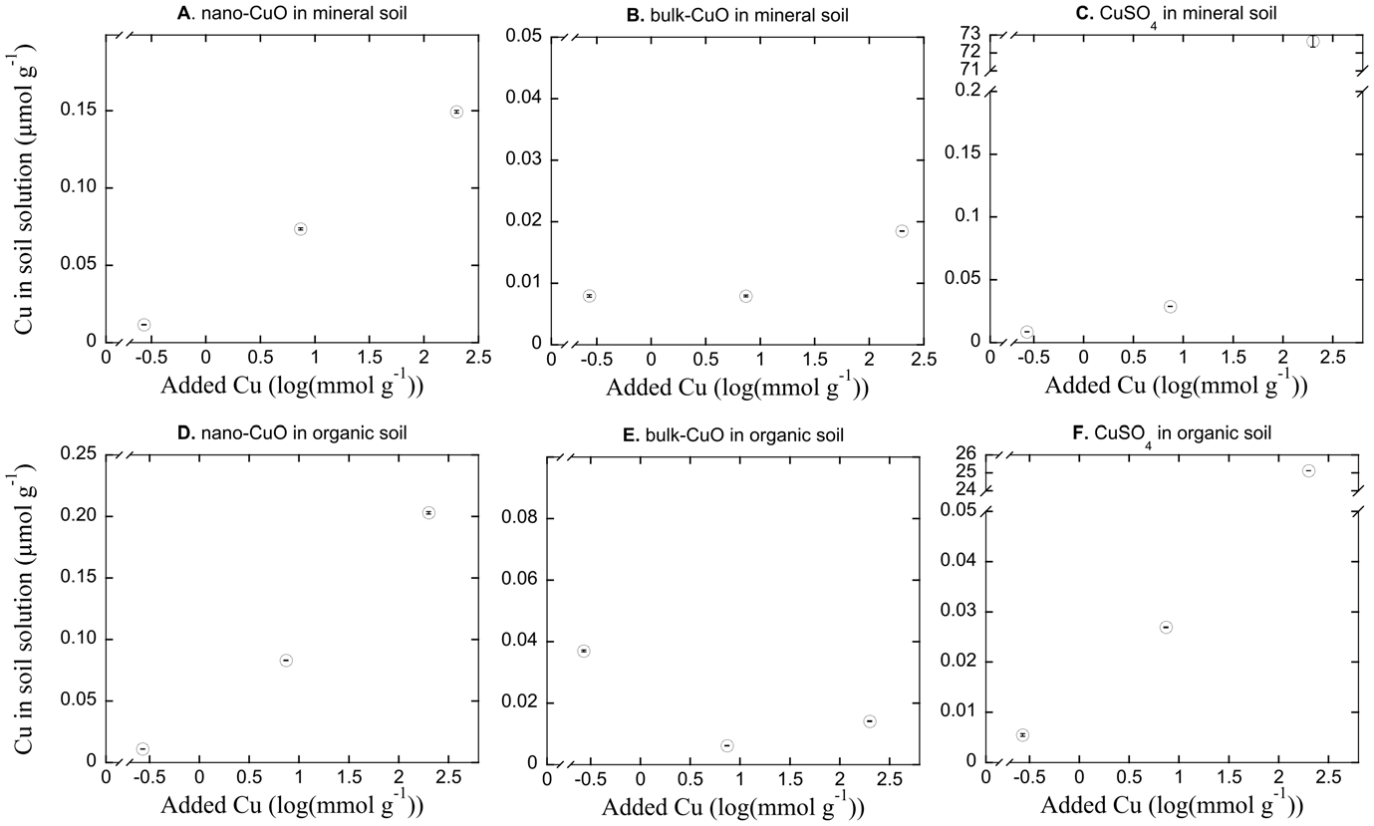
Free Cu in in soil solutions. The relationship between Cu concentration in soil solution and the application rate of nano-CuO (panels A, D), bulk-CuO (panels B, E) and CuSO_4_ (panels C, F) in mineral (panels A, B, C) and organic (D, E, F) soils. Note the broken y-axis scales (panels C, F). Datapoints are the mean of three replicate analyses ±1 SE. Sometimes error bars are hidden by symbols.

The presence of Cu in soil solution, as indicated by the ICP-OES-measurements after a filtration step, increased with higher added concentrations of CuSO_4_ in both the mineral (P<0.001, R^2^>0.99; Fig. 3C) and organic (P<0.001, Fig. 3F) soils. The incremental increases of higher added concentrations appeared to be small below 10 mmol Cu g^−1^, after which the presence of Cu in soil solution increased at a higher rate, suggesting a threshold effect. Further, the solubility of Cu was not complete, and the presence in soil solution only reached maximal levels of around 70 µmol Cu g^−1^ or 20 Cu µmol g^−1^ in mineral and organic soils, respectively, i.e. only a fraction of 1×10^−4^–4×10^−4^ of the added Cu was present in solution. Corresponding measurements showed that the Cu presence following the Bulk-CuO treatment appeared to only at background levels, and no concentration dependence was found in either soil (Fig. 3B, E). Increasing application rates of nano-CuO increased the Cu concentration, as indicated by the ICP-OES-measurements after a filtration step, in soil solution in both soils (P<0.001, R^2^>0.96 for both; Fig. 3A, D), and while the highest concentration of added nano-CuO increased Cu concentration 15 (Fig. 3A) to 20 (Fig. 3D) fold compared to the lowest concentration, to 0.15 and 0.20 µmol Cu g^−1^ in mineral and organic soils, respectively, these levels were less than 1% of those released by CuSO_4_ at the highest concentrations.

### Zn in soil solution

**Figure 4:**
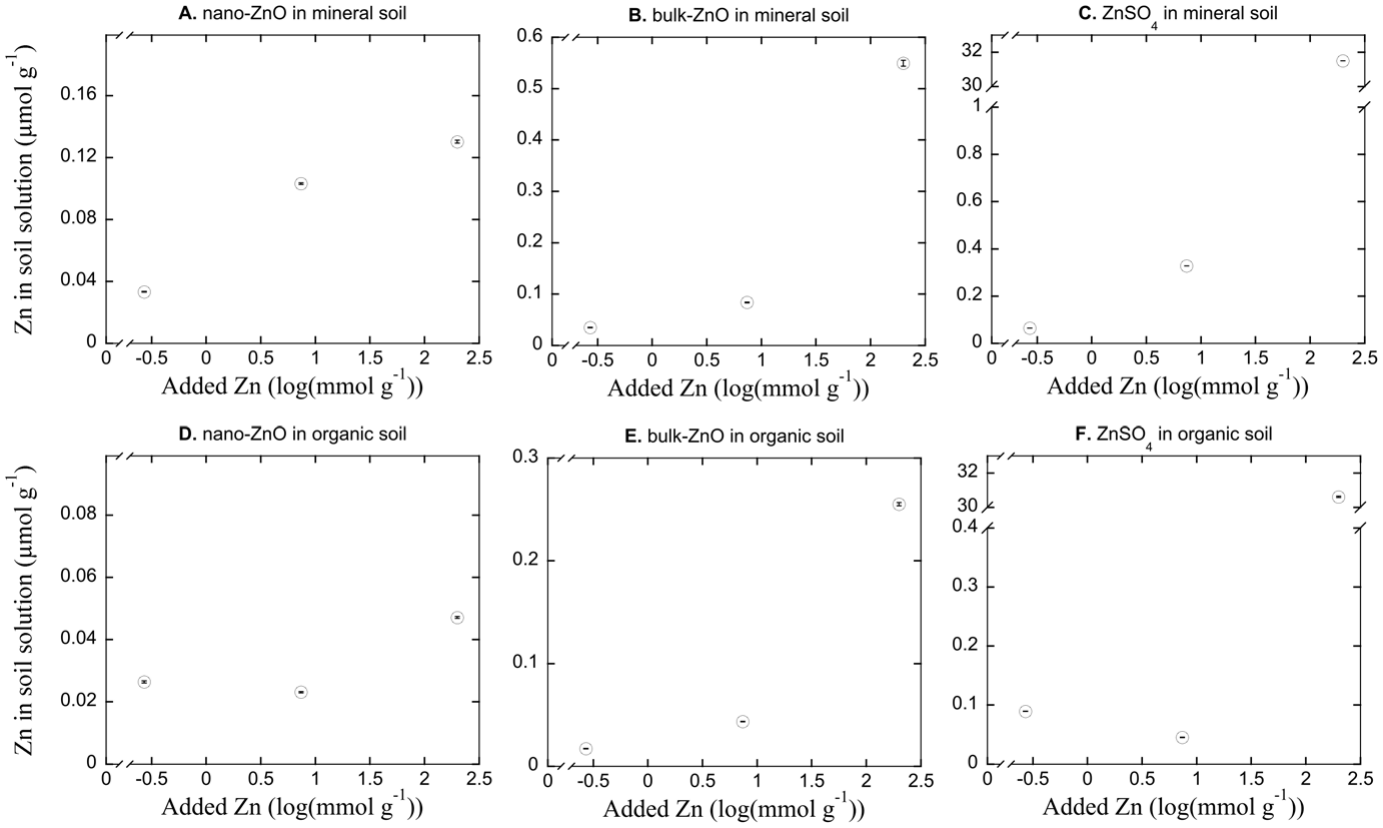
Free Zn in soil solutions. The relationship between Zn concentration in soil solution and the added concentration of nano-ZnO (panels A, D), bulk-ZnO (panels B, E) and ZnSO_4_ (panels C, F) in mineral (panels A, B, C) and organic (D, E, F) soils. Note the broken y-axis scales (panels C, F). Datapoints are the mean of three replicate analyses ±1 SE. Sometimes error bars are hidden by symbols.

The presence of Zn in soil solution, as indicated by the ICP-OES-measurements after a filtration step, increased with higher application rates of ZnSO_4_ in both the mineral (P<0.001, R^2^ = 0.96; Fig. 4C) and organic (P<0.001, R^2^ = 0.95; Fig. 4F) soils. Like for Cu, the incremental increases with higher application rates appeared to be small up to 10 mmol Zn g^−1^, after which the presence in soil solution increased at a higher rate, again suggesting a threshold effect. Further, the solubility of Zn was not complete, and the presence in soil solution only reached maximal levels of around 30 µmol Zn g^−1^ in both soils, i.e. only a fraction of 2×10^−4^ was present in solution. In contrast with Cu, the bulk form of ZnO increased Zn concentrations in soil solution, as measured by the ICP-OES, as shown by clear relationships between application rates and concentration in solution for both mineral (P<0.001; R^2^>0.99, Fig. 4B) and organic (P<0.001; R^2^>0.99; Fig. 4E) soils. The Zn in soil solution increased 10–20 fold between lowest and highest application rates of bulk-ZnO, and reached maximal levels of 0.55 µmol Zn g^−1^ in mineral and 0.25 µmol Zn g^−1^ in organic soils. Nano-ZnO applications resulted in a clear relationship between the estimated concentration in solution and application rate in the mineral soil (P<0.01; R^2^ = 0.94; Fig. 4A) whilst a tendency for the same pattern was also observed in the organic (P = 0.06; R^2^ = 0.63; Fig. 4D) soil. Maximal levels of Zn in solution were just over 0.10 µmol Zn g^−1^ in the mineral soil and less than 0.05 µmol Zn g^−1^ in the organic soil.

### Soil pH effects

Higher concentrations of nano-CuO gradually increased soil pH in both soils, by nearly 1 unit from pH 5.0 to nearly pH 6.0, in the mineral soil (Fig. S1A), and by about 0.3 units, from pH 6.6 to just under pH 7.0, in the organic soil (Fig. S1C). There was very little effect by bulk-CuO on pH in either soil. CuSO_4_ drastically decreased soil pH in both soils, by nearly 2 units, from pH 5.0 to just over pH 3.0 in the mineral soil (Fig. S1A) and by more than 2 units, from pH 6.6 to less than pH 4.0 in the organic soil (Fig. S1C). There was very little influence by nano-ZnO on soil pH in both soils (Fig. S1B, D), while the bulk-ZnO increased soil pH by about 0.5 units in both soils. ZnSO_4_ decreased soil pH by about 2 units in both soils, from pH 5 to just over pH 3 in the mineral soils (Fig. S1B) and from pH 6.6 to about pH 4.5 in the organic soils (Fig. S1D).

### Particle size characterization

Size fractionation showed that 80.8% of the bulk-CuO and 95.5% of the ZnO was made up by particles (or aggregates) larger than 53 µm in at least one dimension.

## Discussion

### Comparative toxicity of nano-CuO

We established a clear dose-response relationship between higher concentrations of nano-CuO and reduced bacterial growth in the mineral soil, and a tendency for reduced growth at the highest concentration of nano-CuO in the organic soil, showing that there was a direct acute toxicity effect acting on soil bacteria. In addition, we could show that macroparticulate (i.e. non-ENP) forms of CuO, bulk-CuO, had no concentration response relationship for bacterial growth. That the ENP form of CuO, nano-CuO, rendered the compound more toxic compared with the bulk form suggested that the toxic effect was directly related to its nano-particulate form. The toxicity of nano-CuO, as well as the relative inertness of bulk-Cu, appeared to be directly related to their dissolution and presence of Cu in solution. While there was no relationship between higher application rates of bulk-CuO and Cu in solution, the dissolved Cu increased with higher application rates of nano-CuO. Comparing the nano-CuO with a soluble form, CuSO_4_, showed that the presence of Cu in solution increased similarly between the compounds up to additions of about 10 mmol Cu g^−1^, but that the CuSO_4_ contributed incrementally more to the dissolved Cu concentration at additions rates beyond this level. This also coincided with a more sharply inhibited bacterial growth in CuSO_4_ treatments at rates higher than 10 mmol Cu g^−1^, further strengthening the connection between measured bacterial toxicity and the presence of Cu in solution. In short, CuO was more toxic in an ENP form than in a macroparticulate (bulk) form, but a more soluble form, CuSO_4_ was yet more toxic. It should be noted, however, that the acidifying effect of higher rates of CuSO_4_ is likely to have added to acute toxicity of the compound, since an unambiguous connection between soil pH and bacterial growth has been established [22], [23]. Further, it has been shown that acute reduction of soil pH by 2 units can reduce soil bacterial growth by about half [24], and additionally, a pH reduction will reduce Cu^2+^ solubility [25], thus affecting the presence of Cu in solution. Thus, it is possible that the acute toxicity of CuSO_4_ is exaggerated by the change in pH in comparison to the oxide forms, while the small positive pH effects by the oxide additions (<0.5 pH-unit alterations between pH 6 and 8 in the different soils) would only be expected to affect bacterial growth negligibly [24]. The consequences of this confounding effect of metal sulphate additions for its property as a positive control are further discussed in following sections.

### Comparative toxicity of nano-ZnO

Similar to the effects of Cu, we determined a clear dose-response relationship between higher concentrations of nano-ZnO and reduced bacterial growth in both soils, again indicating a direct acute toxicity response of the substance on soil bacteria. In addition, we showed that macroparticulate (i.e. non-ENP) forms of ZnO, bulk-ZnO, possessed equally clear or stronger concentration response relationships for bacterial growth, and were even more toxic to bacterial growth (i.e. lower EC_50_ values). As occurred for CuO, the toxicity of both forms of ZnO, appeared to be directly related to their dissolution and presence in soil solution. The dissolved Zn increased with higher application rates of nano-ZnO, but the effect size was rather small. There was an even stronger, or more pronounced, relationship between higher application rates of bulk-ZnO and dissolved Zn. Comparing the nano-ZnO with the bulk-form and the soluble form, ZnSO_4_, showed that the presence of Zn in solution increased similarly for the three compounds up to additions of about 10 mmol Zn g^−1^ in the mineral soil, but that the bulk-ZnO and ZnSO_4_ contributed incrementally more to the Zn concentration in solution than did nano-ZnO with higher additions beyond this rate. In the organic soil the pattern was different, and nano-ZnO or ZnSO_4_ did not clearly increase Zn concentrations in solution with higher application rates up to 10 mmol Zn g^−1^. The bulk-ZnO, in contrast, appeared to contribute to higher concentrations of Zn in solution also up to 10 mmol Zn g^−1^. At higher application rates a threshold was reached for bulk-ZnO and ZnSO_4_, and Zn concentrations in solution were significantly elevated at the highest application rate of ZnSO_4_ compared to bulk-ZnO, and both were many-fold higher than nano-ZnO. The inhibition of bacterial growth correlated intimately with this pattern for Zn in solution, with lowest toxicity (highest EC_50_ values) for nano-ZnO, intermediate for bulk ZnO and highest toxicity (lowest EC_50_) for ZnSO_4_ in mineral soils, while in the organic soil nano-ZnO had lowest toxicity (highest EC_50_), with the ZnSO_4_ being intermediate due to its low contribution to Zn below 10 mmol Zn g^−1^ and bulk-ZnO being most toxic (lowest EC_50_). The toxicity of ZnSO_4_ also had potential to overestimate the toxicity of Zn due to the addition's soil acidifying effect (see discussion for CuSO_4_ above).

### Nano-particulate toxicity

The EC_50_ values determined for the sulphate addition of the metals are well within the span previously obtained for soil organisms, validating our assessments of toxic effects by the additions in general and adding credence to our assessments of the ENP toxicity specifically. Obtained EC_50_ values for the sulphate forms of Cu and Zn were slightly lower than corresponding SIR-estimated levels for soil microorganisms [26] and plants [27] but similar to other assessments using growth-based assays [11], [28], which should be expected given the higher sensitivity of the growth-based assay [14], [15].

The two soils used in this study had very different characteristics. In addition to very different organic matter concentrations, the soils also differed in clay content, pH and cation concentrations. It has been noted that ENPs are highly influenced by the concentration and form of organic matter in soil [29], [30], influencing the ENPs tendency to form aggregates [31] and interaction with biomolecules [32]. Further, it has been suggested that one of the most influential parameters for the toxicity of metal ions, once in solution, is the effective cation exchange capacity [33], largely a product of the soil pH. While, simplistically, it can be assumed that the higher surface area of nano-form compared to bulk-form metal oxides should increase their potential to be dissolved into soil solution, the transition between oxides and free metal ions in solution is a two-stage process. First, metal oxide particles may interact with particles and organic matter constituents of the soils [34], e.g. forming aggregates, affecting their effective surface area in the soil. Second, once dissolved into soil solution, the metals will interact with other ions in solution and electronegative or charged functional groups on solids or macromolecules, forming metal complexes, which can reduce their concentration as free ions in solution, affecting the mass balance. The resulting contribution by metal additions to metal ion presence in soil solution is, consequently, hard to predict generally [35]–[37], and especially hard for ENP forms [29], [31]. Thus, we safely can conclude that the presence of the metals in solution was differentially affected between the three different forms of Zn and Cu added in the two soils, but we are not able to assign these differences to specific factors such as organic matter content or soil pH, and more systematic comparisons of ranges of soils are required before we can start assigning these differences to mechanisms.

Using metal sulphates proved to be imperfect control treatments to evaluate the toxic effects of soluble metals. Both Cu and Zn sulphates greatly reduced the soil pH by up to 2 units in the highest metal addition rates, a well-known property of metal sulphate additions [38]. It is likely that this extensive acidification added to the toxic effects of the metal sulphates, and the presence of protons in solution is also known to modulate metal toxicity by competing with metal cations for biotic binding sites [39], [40], [41]. Further, higher solubility of metals at reduced pH, with acidification commencing and quickly increasing at 10 mmol metal g^−1^ and beyond, could have contributed the threshold-like effect observed for metals in solution (Fig. 3, 4). However, the comparative nature of our experiment design allows for the evaluation of the putative confounding toxicity that the acidification affected our bacterial growth based toxicity assay with. While the sulphide form of Zn consistently decreased the pH in both soil types by about 2 units, the bulk-ZnO had negligible effects on pH (supplementary fig. S1). A mechanism of toxicity shared by the substances, on the other hand, were bacterial exposure to Zn. However, the bulk-ZnO proved more toxic to bacteria, and reduced bacterial growth to levels comparable to ZnSO_4_ at the highest application rates. This suggests that while pH in principle could have added to the metal toxicity, there is no evidence from our data to suggest that it did so to an important degree, but rather that the metal itself exerted the toxic effect. This result was also consistent for both soils types. The same argument would also be applicable to evaluating putative osmotic effect and ionic strength effects of the metal sulphate additions [42], where this also would have acted to make the salt form, ZnSO_4_, more toxic than bulk-ZnO.

Three candidate hypotheses for the putative toxicity of ENP forms of metal oxides have been forwarded: (i) generation of reactive oxygen species that can cause lipid peroxidation and disrupt cell membranes as well as damage DNA [43], [44], (ii) membrane disorganization [45] and (iii) release of metal ions [44], [46] of well known ecotoxicity [33], [47]. While we are not able to pinpoint the precise reasons for how the different forms of metal oxides and sulphates contributed to Cu and Zn concentrations in soil solution, we found a robust and unambiguous connection between bacterial growth inhibition and the measured metal concentration in soil solution. Our estimate of metal concentrations in soil solution is not capable of resolving in what form it is present there, as metal ions, suspended ENPs or dissolved metal complexes. All we can assess is the total metal concentrations (with a particulate size <0.45 µm due to the filtration step) carried in solution. If our additions resulted in ENP presence in soil solution after addition to soil samples, and these contributed to toxicity, detectable metal concentrations in the nano-Cu and nano-Zn treatments would be expected to expose bacteria to a higher toxicity than similar concentrations of other forms (bulk and sulphate forms). There is no such evidence in our data, and the toxicity of metal concentrations in soil solution, irrespective of source (ENP or not), were found to inhibit bacteria to the same extent. Although we are not able to explicitly rule out that nano-particle related generation of reactive oxygen species or disorganization of membranes contributed to the toxicity of the ENPs, our results are consistent with only one of these mechanisms being influential for bacterial growth inhibition – the contribution to metal ion concentration in soil solution. Thus, the most parsimonious interpretation of our results would be that the contribution by the added metals to metal ion concentrations in soil solution was the more important mechanism for the observed toxicity to bacterial growth in soils, and that the direct influence of nano-particles on bacteria was negligible beyond this. While this conclusion is built on conjecture, direct measurements of metal ions concentrations, by means of e.g. a Cu specific electrode to measure Cu^2+^ [48], [49], could be used to confirm the causality in our interpretation, and suggests a way forward.

That metal exposure can be detrimental for soil biota has been well-known for decades [42] and heavy metal toxicity is growing to be a mature subject field [33], as evidenced by the development of the biotic ligand model (BLM) to predict environmental toxicity of Cu, Zn and other metals [39], [40], [41]. The increasing application of ENP forms of metals have resulted in a new surge in studies of metal ENP [2], [5], and assessments of ENP metal oxides in soil have been able to determine and show clear toxicity to e.g. soil bacteria [7]. However, to date, there is a shortage of careful evaluations of how the ENP form of the metal, *per se*, modulates its toxicity. We show that ENP oxides of Zn and Cu can inhibit bacterial growth in different soils. Moreover, the toxicity of the ENP metal oxide form differed from the non-ENP, but the difference was contingent on the soil studied. More soluble forms (sulphates) of Cu and Zn proved more toxic to soil bacteria than the metal oxide forms, ENP or otherwise. Emerging from these results, we find a tight connection between the presence of metal in soil solution and the resulting toxicity of the added metals, suggesting that the ENP form can be more toxic than non-ENP forms, but only when dissolution of the metal is higher in this form (this is the soil dependence). Although a framework to assess bioavailable metal concentrations has proved elusive [33], [36], [37], we suggest that efforts toward the synthetic goal of e.g. BLM [39], [40], [41] are more valuable than spending resources to reassess ecotoxicology of metal ENPs separately from general ecotoxicology of metals.

## Supporting Information

Figure S1 The relationship between the pH in soil solution and the added concentration of nano-CuO, bulk-CuO and CuSO_4_ (panels A, C) and nano-ZnO, bulk-ZnO and ZnSO_4_ (panels B, D) in mineral (panels A, B) and organic (C, D) soils. Datapoints are the mean of two replicate analyses ±1 SE. Sometimes error bars are hidden by symbols.
(TIF)
