# Supplementary figures and images for: Comparative Toxicity of Nanoparticulate CuO and ZnO to Soil Bacterial Communities
Source: PLoS One. 2012 Mar 29;7(3):e34197. doi: 10.1371/journal.pone.0034197 (PMC3315546; doi:10.1371/journal.pone.0034197)

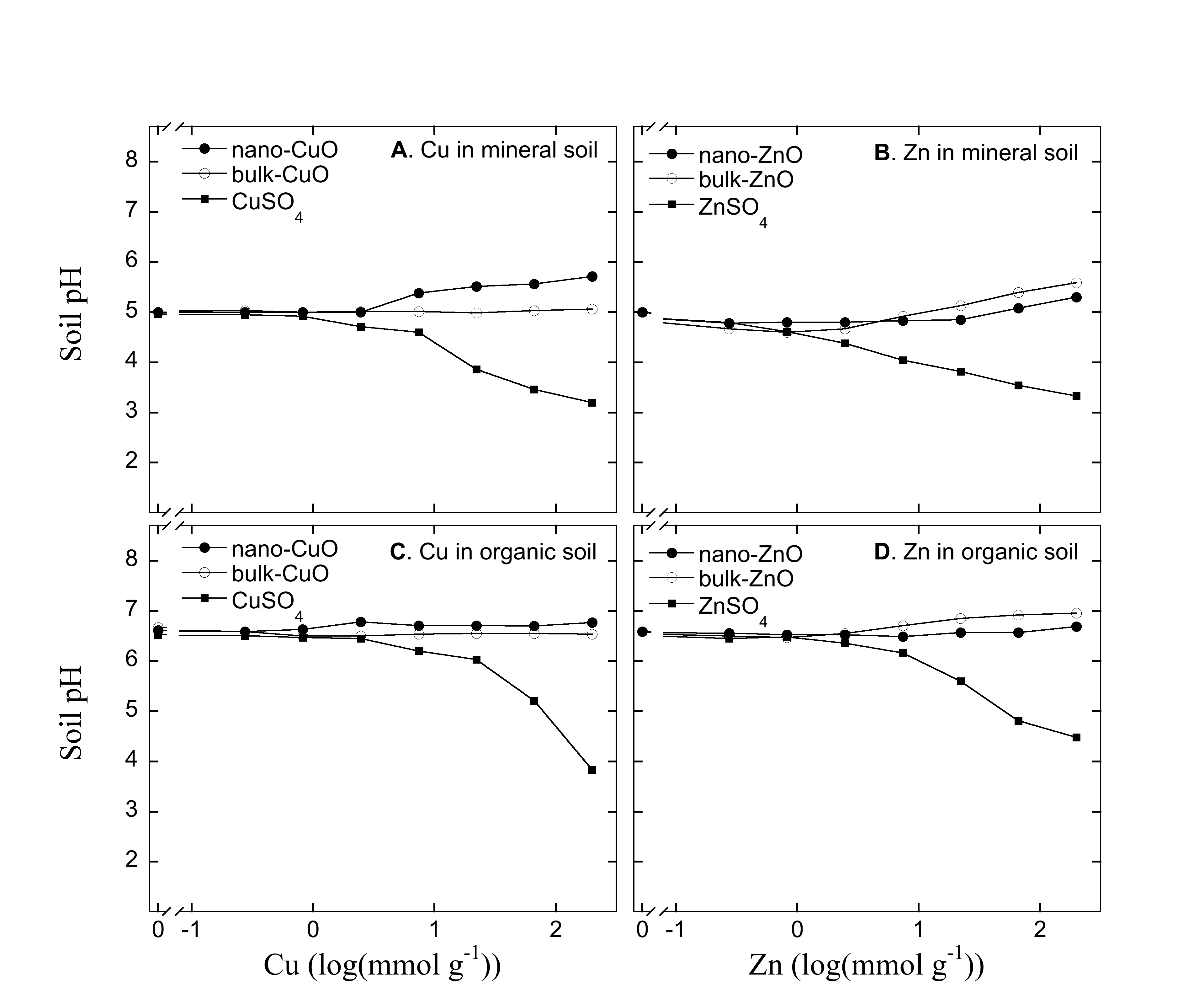

Supplement: Figure S1 — The relationship between the pH in soil solution and the added concentration of nano-CuO, bulk-CuO and CuSO4 (panels A, C) and nano-ZnO, bulk-ZnO and ZnSO4 (panels B, D) in mineral (panels A, B) and organic (C, D) soils. Datapoints are the mean of two replicate analyses ±1 SE. Sometimes error bars are hidden by symbols. (TIF) [file pone.0034197.s001.tif]
